# Foetal haemoglobin and the dynamics of paediatric malaria

**DOI:** 10.1186/1475-2875-11-396

**Published:** 2012-11-28

**Authors:** Erica MW Billig, Philip G McQueen, F Ellis McKenzie

**Affiliations:** 1 National Institutes of Health, Fogarty International Center, Building 16, Room 303, Bethesda, MD, 20892, USA; 2 National Institutes of Health, Center for Information Technology, 12 South Drive, Bethesda, MD, 20892, USA

**Keywords:** Malaria, *Plasmodium falciparum*, Foetal haemoglobin

## Abstract

**Background:**

Although 80% of malaria occurs in children under five years of age, infants under six months of age are known to have low rates of infection and disease. It is not clear why this youngest age group is protected; possible factors include maternal antibodies, unique nutrition (breast milk), and the presence of foetal haemoglobin (HbF). This work aims to gain insight into possible mechanisms of protection, and suggest pathways for focused empirical work, by modelling a range of possible effects of foetal haemoglobin and other red blood cell (RBC) developmental changes on parasite dynamics in infants.

**Methods:**

A set of ordinary differential equations was created to investigate the leading hypotheses about the possible protective mechanisms of HbF-containing red blood cells, in particular whether HbF suppresses parasite population growth because parasite multiplication in individual RBCs is lower, slower or absent. The model also incorporated the intrinsic changes in blood volume and haematocrit that occur with age, and the possibility of parasite affinities for HbF-containing RBCs or reticulocytes.

**Results:**

The model identified several sets of conditions in which the infant remained protected, or displayed a much slower growth of parasitaemia in the first few months of life, without any intervening immune response. The most protective of the hypothesized mechanisms would be the inhibition of schizont division in foetal RBCs so that fewer merozoites are produced. The model showed that a parasite preference for HbF-containing RBCs increases protective effects for the host, while a preference for reticulocytes has little effect.

**Conclusions:**

The results from this simple model of haematological changes in infants and their effects on *Plasmodium falciparum* infection dynamics emphasize the likely importance of HbF and RBC number as an explanatory factor in paediatric malaria, and suggest a framework for organizing related empirical research.

## Background

Malaria disproportionately affects children under five years of age [1]. Repeated infection builds immune responses that protect against severe clinical disease and reduce parasitaemia [2-4], and, presumably, older children in endemic areas have been exposed more often and to a wider range of antigens than younger children. Hence age is often considered a surrogate for cumulative exposure, and age differences in response are attributed accordingly. There may be more to the explanation, however, given that intrinsic developmental differences between children under five and older children, or adults, may affect responses to malaria infection [5,6]. Among the developmental age differences that may affect the course and outcome of a malaria infection are the age-specific abundance and properties of red blood cells (RBCs).

A human malaria infection begins when the parasite enters the bloodstream in the saliva of an infectious, biting mosquito. The parasite invades the liver, multiplies there for approximately 10 days, and then releases thousands of merozoite forms into the bloodstream. Each merozoite invades a RBC, multiplies, and – if *Plasmodium falciparum* or *Plasmodium vivax*, the most common species – in about 48 hours bursts the RBC to release eight to 32 new merozoites, which invade new RBCs and continue the cycle [7]. Virtually all of the pathology of human malaria is associated with this cycle in RBCs, which continues until the host mounts an immune response sufficient to control the infection, or the host dies.

Presumably, the basic biological process of parasite invasion and multiplication in RBCs is independent of the literal age of a host (i e, the parasite does not “know” how old its host is), and so, whatever the host’s age, cycles proceed roughly as sketched above, with regular numbers of merozoites released at each cycle, in cycles of regular length. The total blood volume of a host increases very significantly with age and size [8,9]. Therefore, all else equal, overall parasitaemia will increase more rapidly in hosts with smaller total numbers of RBCs – younger children – and the consequent anaemia will become more severe more quickly. That is, while the RBC count of 4–6 × 10^6^ ml^-1^ of blood may be similar between infants and adults, the blood volume of an adult is 10–15 times that of an infant, so any given population of parasites in an adult host will represent a much lower total parasitaemia than in an infant host.

Another notable difference between infants and older children is that a much higher proportion of RBCs in infants contain foetal haemoglobin (HbF) rather than adult haemoglobin (HbA). HbF is produced only in humans, apes and Old World monkeys: it has a stronger affinity for oxygen than HbA, and so facilitates oxygen transfer across the placenta from mother to foetus during gestation [10]. Beginning in the final trimester of pregnancy, and continuing into the first months of life, haemoglobin production usually largely switches from HbF to HbA, and the fraction of RBCs that contains HbF declines accordingly. The fraction of RBCs that contains HbF at birth varies from about 50% to 100% among neonates [11,12]. Variation may be related to differences in gestation period and/or in the complex polygenic control of haemoglobin production [13-15]. In most adults only about 1% of RBCs contain HbF, but in those with hereditary persistence of foetal haemoglobin (HPFH), a benign condition in which mutations or deletions in the β- or γ-globin genes or regulatory regions alter normal haemoglobin switching, 10-100% of RBCs contain HbF.

Infants under six months of age have a low rate of infection and incidence of severe disease compared to older children [16], despite the relative immaturity of their immune systems. Several factors may contribute to this protection, including passively transferred maternal antibodies, unique nutrition (breast milk), and elevated HbF [17]. Current evidence suggests that HbF retards the expansion of a *Plasmodium* population because *Plasmodium* parasites do not survive as well in HbF-containing RBCs as in those containing HbA [18-23], though details and specific mechanisms remain unclear. There could be several reasons for the retardation: the HbF cell may be a “dead end,” meaning that the parasite can invade but cannot replicate, or cannot escape, and so dies within the RBC. Alternatively, the growth process may be suppressed or slower in HbF-containing RBCs, such that fewer merozoites are released per infected RBC, or they take longer to develop to the point of bursting. In addition, *P. falciparum* may have a higher affinity for HbF-containing RBCs than HbA-containing RBCs. *P. falciparum* may also have (like *P. vivax*) a higher affinity for reticulocytes – the youngest age class of RBCs – which may be present in higher proportions in growing infants, and so may affect infection dynamics. In any case, the proportion of HbF-containing RBCs may be a factor in the protection of infants from severe clinical disease and high parasitaemia: by slowing parasite population growth, they may provide extra time for an effective response by the infant’s developing immune system or maternal antibodies.

Although several possible factors underlying variable protection in infants have been suggested, including the effects of maternal antibodies, nutritional differences, and HbF, none has yet been confirmed. Foetal haemoglobin is one of a number of mechanisms, reviewed in an earlier paper [6], that could help to explain the observed neonatal protection from severe disease. Infants have immature immune systems, in which key elements are developing asynchronously [6], so any added protective factors are likely to be of particular importance. This is a difficult set of topics to study, however, given the age group and multiple confounding factors.

No mathematical model to date has investigated the effects of any developmental age difference on a malaria infection. Here previous models of RBC dynamics in malaria infections are extended to focus specifically on the effects of lower RBC counts and higher HbF proportions on the dynamics of *P. falciparum* infections in infants [24-26].

## Methods

A set of ordinary differential equations (ODEs) was used to model the dynamics of circulating RBCs, including the effect of infant growth on total blood volume (via the rate of growth of erythropoietic tissue), the switch in production of HbF- to HbA-containing RBCs, and the change in haematocrit in the first few months of life. The dynamics of two RBC lines were modelled – the uninfected (1) foetal and (2) adult erythrocytes – then the dynamics of malaria infection were incorporated into the dynamics of the circulating RBCs. Three main populations of *Plasmodium* parasites are involved in the pathology of malaria in an infant: (1) those in infected foetal erythrocytes, (2) those in infected adult erythrocytes, and (3) free merozoites in the blood. (Gametocytes and any cryptic sexual forms were ignored in the model.) Thus, the dynamical model considered has five separate populations, describing the dynamics of uninfected and infected foetal (HbF) RBCs, uninfected and infected adult (HbA) RBCs, and free merozoites within an infant host. For conciseness, vector and matrix notation (Additional file 1) are used to describe the dynamical equations.

### Basic structure of the dynamical model

Consider an *N* × *1* vector **P**, the components of which may evolve in time. Define *T*(**P**) to be the sum of the components of **P**. Let the following ordinary differential equation (ODE) system in time *t* determine the evolution of the components:

(1) $$\text{d}P/dt=S\left(t\right)\delta \left(1\right)-\Lambda \;D\;P$$

where *S(t)* is greater than or equal to zero for all *t*, Λ > 0 and dΛ = 0. The components of the vector **δ**(1) are zero except for the first one. Matrix ***D*** is sparse with all diagonal components = 1, and all components just below the diagonal = −1; see Additional file 1 for details. One can show that the contribution to –d*T* (**P**)/dt from *S* at time *t* - Δ*t* is approximately a Gaussian function of Δ*t* with a mean *D* = *N* Λ^-1^ and standard deviation σ = *D N*^-1/2^[24,27]. The ODE system described by equation (1) models the dynamics of individual organisms with total population *T*(**P**) and source term *S(t)*. If individuals age with an average lifespan of *D* with standard deviation σ, then knowing the tangible quantities *D* and σ sets the abstract quantities *N* and Λ. (If *D* = σ, the ODE system reduces to one of simple exponential decay with a source term.) Although the vector–matrix notation is introduced for brevity, **P** itself can be thought of as containing all the information about the “state” of the population possible in this model. The matrix Λ ***D*** operates on the state vector **P**, incorporating the contribution of the source *S(t)* into the time evolution of **P**. This formalism may seem complicated initially, but it incorporates the non-instantaneous propagation of changes in the source *S(t)* into the population. One could think of other formalisms that incorporate time delays and dispersion in aging, but this particular one allows the use of efficient ODE solvers.

### Total blood volume

In uninfected individuals, RBCs containing HbA circulate for ~120 days, while RBCs containing HbF circulate for ~70 days, at which point the RBC is cleared by the spleen [28]. In adults, there are ~5 × 10^6^ RBCs per μl of blood. In neonates, the haematocrit dips within the first few months of life, and then rises again to adult levels [29]. Presumably, this transient dip is related to changes in RBC production in combination with the growing mass, and thus total blood volume, of the infant.

Since RBC and parasite populations are assessed empirically as cell counts per μl of blood, the model dynamics were formulated to consider the population per unit volume of blood rather than the total population in the host. However, the volume growth of the child must be carefully incorporated. If the state of a population of a parasite or RBC line is given by **P**, then consider **ρ** = *V*_B_^-1^*X***P**, where *V*_B_ is the blood volume of the host. (For simplicity, uniform mixing in the blood is assumed.) The time evolution of **ρ** is given by

(none1) $$\begin{array}{c} \text{d}\rho /\text{dt}=\text{d}\left({V_{\text{B}}}^{-1}\right)/\text{dt}\;P+{V_{\text{B}}}^{-1}\\ \text{d}P/\text{dt}=s(t)\delta \left(1\right)–(\Lambda D+\Lambda_{\text{V}}I)\rho \end{array}$$

where

(none2) $$s\left(t\right)={V_{\text{B}}}^{-1}S\left(t\right)$$

and

(2) $$\Lambda_{\text{V}}={V_{\text{B}}}^{-1}\text{d}\left(V_{\text{B}}\right)/\text{dt}$$

The sum of the components of **ρ**, *T(***ρ***)*, is *V*_B_^-1^*X T(***P***)*, the density of the population in the blood. So from the point of view of population density, the growth of blood volume contributes a decay factor Λ_V_: a growing volume tends to dilute the concentration of cells. Note that Λ_V_ itself is time dependent, as the rate of volume growth slows as the child ages.

### Erythrocyte source

Near the time of birth, haemoglobin production usually switches from predominantly HbF to predominantly HbA. The timing of this switch, and the rate and degree of change, vary from infant to infant. The levels of circulating HbF usually reach adult levels of 1 – 2% around one year of age. The production of foetal erythrocytes per kilogram of mass of the host decreases nearly exponentially with age (*t*) as the host ages, while the production of adult erythrocytes per kilogram of mass of the host is a sigmoid-like function of *t* that reaches a maximum some months after birth. (For a review, see [13]) The time evolution of the erythrocyte sources in equation (5) is modelled by

(3) $$\begin{array}{l} ESa\left(t\right)=Pamx\;X\text{tanh}((t+t_{\text{OSa}})/{\text{t}}_{\text{Ca}})X\;W\left(t\right)\;V_{\text{B}}{\left(t\right)}^{-1}\\ ESf\left(t\right)=(Pfmx\;X\text{exp}(-\text{t}/{\text{t}}_{\text{Cf}})+Pfmn)X\;W\left(t\right)\;V_{\text{B}}{\left(t\right)}^{-1} \end{array}$$

Here *W(t)* is the mass of the host at age *t*. The time evolution of *W(t)* and *V*_B_*(t)* is discussed in Additional file 1. The values of the parameters *Pamx*, *t*_OSa_, τ_Ca_, *Pfmx*, τ_Cf_ and *Pfmn* are given in Table 1. Three pairs of time constants were used to model different changes in the rates of HbF and HbA production (Table 1). Using these parameters, the HbF:HbA ratio reaches 1:1 at 65, 97, and 163 days of age.

**Table 1:** Hematological parameters

**Parameter**	**Symbol**	**Value**
Maximum adult reticulocyte production rate per kilogram of tissue	*Pamx*,	1.21 × 10^8^ (kg hr) ^-1^
Maximum foetal reticulocyte production rate per kilogram of tissue	*Pfmx*	1.7 × 10^8^ (kg hr) ^-1^
Minimum foetal reticulocyte production rate per kilogram of tissue	*Pfmn*	2.48 × 10^6^ (kg hr) ^-1^
Time offset for production of adult erythrocytes	*t*_OSa_	50 days
Time constant for production of adult erythrocytes	τ_Ca_	75, 150, and 300 days
Time constant for decay of production of foetal reticulocytes	τ_Cf_	50, 100, and 200 days
Volume of adult red blood cell	*V*_a_	8.0 × 10^-8^ μm^3^
Volume of foetal red blood cell	*V*_f_	1.25 × 10^-7^ μm^3^

Baseline parameters for adult (HbA) and foetal (HbF) RBCs [44].

As a measure of the health of the host, the haematocrit *hc* was tracked, calculated as

(4) $$hc=100\%X\left(V_{\text{a}}\left(T\left(Rea\right)+T\left(Ma\right)\right)+V_{\text{f}}\left(T\left(Ref\right)+T\left(Mf\right)\right)\right)$$

Here *V*_f_ and *V*_a_ are the mean volumes of foetal and adult erythrocytes. Their values are given in Table 1. **Rea** and **Ma** are the population state vectors for the reticulocytes and mature RBCs for adult RBCs and Ref and Mf are the corresponding state vectors for the foetal RBCs; see next subsection. If the value of *hc* drops under 24%, it is assumed that the host dies.

### Red blood cell populations

Some evidence indicates that *P. falciparum* preferentially invades young RBCs – reticulocytes – regardless of haemoglobin content [19,30], and also that *P. falciparum* preferentially invades HbF-containing RBCs [21]. It remains uncertain whether *P. falciparum* in fact prefers HbF-containing RBCs, or, given the shorter lifespan and changing production patterns in the first few months of life, the key factor is that HbF-containing RBCs have a younger age distribution, skewed toward reticulocytes. The ability to test these possibilities was incorporated into the model by creating a reticulocyte subgroup and a mature subgroup for both the foetal and the adult RBC populations. The model incorporates the effects of the switch from the production of foetal erythrocytes to adult erythrocytes, as well as the increase in blood volume as the host ages. The equations that describe the dynamics of the RBC populations are:

(5) $$\begin{array}{c} \text{d}Rea/\text{dt}=ESa(t)\delta (1)-(\Lambda_{\text{Rea}}D+(\varsigma_{\text{Rea}}\mu +\Lambda_{\text{V}})I)Rea\\ \text{d}Ma/\text{dt}=\Lambda_{\text{Rea}}L(Rea)\delta (1)-(\Lambda_{\text{Ma}}D+(\varsigma_{\text{Ma}}\mu +\Lambda_{\text{V}})I)Ma\\ \text{d}Ref/\text{dt}=ESf(t)\delta (1)-(\Lambda_{\text{Ref}}D+(\varsigma_{\text{Ref}}\mu +\Lambda_{\text{V}})I)Ref\\ \text{d}Mf/\text{dt}\;=\Lambda_{\text{Ref}}L(Ref)\delta (1)-(\Lambda_{\text{Mf}}D+(\varsigma_{\text{Mf}}\mu +\Lambda_{\text{V}})I)Mf \end{array}$$

Here, ***I*** is the identity matrix and *ESa(t)* and *ESf(t)* are the source rates of production of adult and foetal reticulocytes, respectively, per unit volume of blood. These quantities change as the host ages, in a manner specified below. ζ_Rea_, ζ_Ma_, ζ_Ref_, and ζ_Mf_ are the binding affinities of the merozoites to the respective RBCs; the values are defined in the next section. The state vector of the merozoites is **μ**. The rates Λ_Rea_, Λ_Ma_, Λ_Ref_, Λ_Mf_ and the vector lengths were defined as in the subsection “Basic Structure of Dynamical Model” using the values specified in Table 2.

**Table 2:** Populations used in the model

**Population**	**State vector**	**Average duration (h)**	**Standard deviation (h)**
Merozoites	**μ**	*D*μ = 0.1	σ μ = 0.1 = *D*μ
Ring stage in adult RBC	**Ra**	*D*_Ra_ = 12	σ_Ra_ = 1.2
Early trophozoite in adult RBC	**Ea**	*D*_Ea_ = 12	σ_Ea_ = 1.2
Late trophozoite in adult RBC	**La**	*D*_La_ = 12	σ_La_ = 1.2
Schizont in adult RBC	**Sa**	*D*_Sa_ = 12	σ_Sa_ = 1.2
Ring stage in foetal RBC	**Rf**	*D*_Rf_ = 12	σ_Rf_ = 1.2
Early trophozoite in foetal RBC	**Ef**	*D*_Ef_ = 12	σ_Ef_ = 1.2
Late trophozoite in foetal RBC	**Lf**	*D*_Ef_ = 12	σ_Lf_ = 1.2
Schizont in foetal RBC	**Sf**	*D*_Sf_ = 12 or 72	σ_Sf_ = 0.1
Adult Reticulocyte	**Rea**	*D*_Rea_ = 36	σ_Rea_ = 6
Adult mature RBC	**Ma**	*D*_Ma_ = 2844	σ_Ma_ = 168
Foetal Reticulocyte	**Ref**	*D*_Ref_ = 36	σ_Ref_ = 6
Foetal mature RBC	**Mf**	*D*_Mf_ = 1644	σ_Mf_ = 120

Durations of intracellular parasite stages in adult (HbA) and foetal (HbF) RBCs [45]. Merozoite duration [46], Adult erythrocyte durations: [47], Foetal erythrocyte durations [28]. The standard deviations are plausible guesses.

### Parasite populations

Five morphologically distinct populations of asexual parasite cells were considered: (1) ring stage, (2) early trophozoites, (3) late trophozoites, (4) schizonts, and (5) merozoites. The possibility is allowed that the parasite can develop in either foetal or adult erythrocytes, but possibly at different rates of development and with different efficiencies of reproduction in each type. The list of populations as well as the average durations of individual residence in those populations, along with the standard deviations in the durations, is given in Table 2.

The primary release of merozoites from the liver apparently involves the release of thousands of merozoites in clusters [31]. For simplicity it is assumed that primary release occurs over a duration τ_PR_ = 24 hr with total of 10^5^ merozoites released into the blood volume *V*_B_ at a constant rate per unit volume κ_PR_ = 10^5^ (24 h *X V*_B_)^-1^. The average number of merozoites subsequently released by bursting schizonts of *P. falciparum* in human blood culture is known from direct microscopy to be >16 [32], so in this report the average number of merozoites released from a bursting schizont formed in an adult erythrocyte is taken to be *p*_a_ = 16. The possibility is allowed that the average number of merozoites released from a bursting schizont formed in a foetal erythrocyte, *p*_f_, may be different from *p*_a_. The binding affinity of merozoites to target RBCs is inferred from observations of parasite growth in neurosyphilis patients treated for malaria therapy [33,34], and inoculated volunteers [35], to be 10^-6^ – 10^-5^ μl hr^-1^. Here, ζ_Ma_, the binding affinity of a merozoite to the mature adult RBC, is taken to be 3.33333 × 10^-6^ μl hr^-1^. The possibility is allowed that the merozoite binding affinity to adult reticulocytes, ζ_Rea_, mature foetal RBCs, ζ_Mf_, and foetal reticulocytes, ζ_Ref_, may differ from ζ_Ma_. The value is only changed when directly stated.

Using the information above, letting *t* be the time since the birth of the infant, and taking *Ag*_PR_ as the age of the infant when primary release starts, the ODE system for the parasite populations is:

(6) $$\begin{array}{c} \text{d}\mu /\text{dt}=\kappa_{\text{PR}}\Theta \left(t-Ag_{\text{PR}}\right)\Theta (Ag_{\text{PR}}-\tau_{\text{PR}}-t)+p_{\text{a}}\Lambda_{\text{Sa}}Sa_{\text{NSa}}+p_{\text{f}}\Lambda_{\text{Sf}}Sf_{\text{NSf}}\\ -(\varsigma_{\text{Rea}}T(Rea)+\varsigma_{\text{Ma}}T(Ma)+\varsigma_{\text{Ref}}T(Ref)+\varsigma_{\text{Mf}}T(Mf)+\Lambda \text{μ}+\Lambda_{\text{V}})\mu \\ \text{d}Ra/\text{dt}=(\varsigma_{\text{Rea}}T(Rea)+\varsigma_{\text{Ma}}T(Ma))\mu \;\delta (1)-(\Lambda_{\text{Ra}}D+\Lambda_{\text{V}}I)Ra\\ \text{d}Ea/\text{dt}=\Lambda_{\text{Ra}}L(Ra)\delta (1)-(\Lambda_{\text{Ea}}D+\Lambda_{\text{V}}I)Ea\\ \text{d}La/\text{dt}=\Lambda_{\text{Ea}}L(Ea)\delta (1)-(\Lambda_{\text{La}}D+\Lambda_{\text{V}}I)La\\ \text{d}Sa/\text{dt}=\Lambda_{\text{La}}L(La)\delta (1)-(\Lambda_{\text{Sa}}D+\Lambda_{\text{V}}I)Sa\\ \text{d}Rf/\text{dt}=(\varsigma_{\text{Ref}}T(Ref)+\varsigma_{\text{Mf}}T(Mf))\mu \;\delta (1)-({\text{L}}_{\text{Rf}}D+\Lambda_{\text{V}}I)Rf\\ \text{d}Ef/\text{dt}=\Lambda_{\text{Rf}}L(Rf)\delta (1)-(\Lambda_{\text{Ef}}D+\Lambda_{\text{V}}I)Ef\\ \text{d}Lf/\text{dt}=\Lambda_{\text{Ef}}L(Ef)\delta (1)-(\Lambda_{\text{Lf}}D+\Lambda_{\text{V}}I)Lf\\ \text{d}Sf/\text{dt}=\Lambda_{\text{Lf}}L(Lf)\delta (1)-(\Lambda_{\text{Sf}}D+\Lambda_{\text{V}}I)Sf \end{array}$$

(Here, Θ (*x*) = 1 if *x* > 0, zero otherwise.)

Note that since *D*μ = σμ, the state vector for the merozoites, **μ**, has only one component, so it was treated it as a scalar in equation (6). The rates Λμ, Λ_Ra_, Λ_Ea_, Λ_La_, Λ_Sa_, Λ_Rf_, Λ_Ef_, Λ_Lf_, Λ_Sf_ and the vector lengths were defined as in the subsection “Basic Structure of Dynamical Model,” using the values specified in Table 2.

For further details on the Methods, see Additional file 1.

## Results

**Figure 1:**
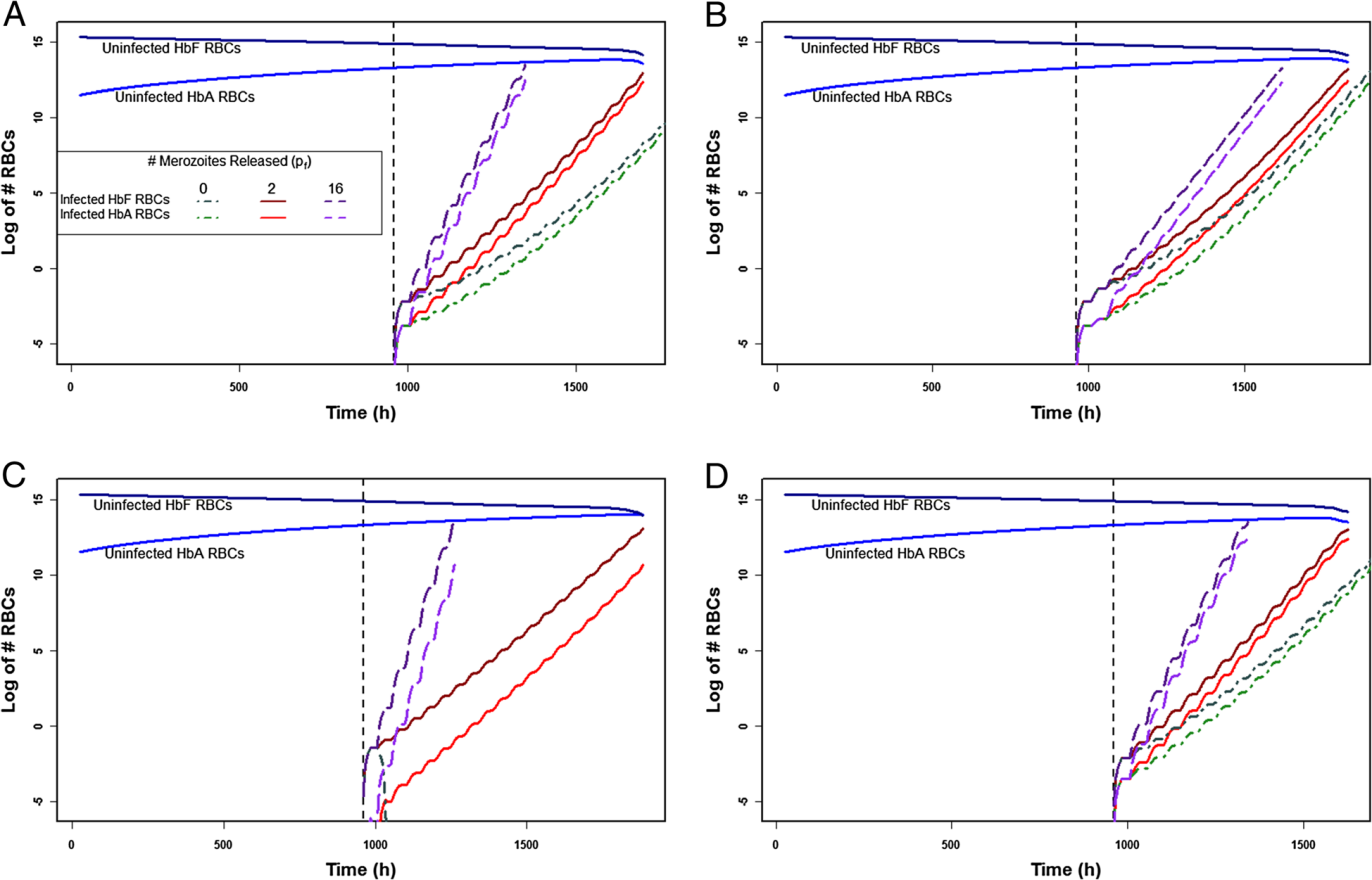
**The number of uninfected and infected HbA- and HbF-containing RBCs (y-axis) in an infant infected at 40 days of age (x-axis: 1000 h).** Each graph shows the rate of parasite population growth with p_f_ = 0, 2, or 16. The number of uninfected RBCs is shown for p_f_ = 2: there is no evident difference in these numbers with p_f_ = 0 or p_f_ = 16. **A**: Parameters set to values in Tables 1 and 2; **B**: 108 h life cycle (D_Sf_ =72); **C**: Parasite has a stronger affinity for HbF-containing RBCs (ζ_Ref_ = 10ζ_Rea_ and ζ_Mf_ = 10ζ_Ma_); **D**: Parasite has a stronger affinity for reticulocytes (ζ_Ref_ = 10ζ_Mf_ and ζ_Rea_ = 10ζ_Ma_).

Because little is known about the protective effects of HbF at the single-RBC level, several hypotheses about the failure of *P. falciparum* to develop normally within an HbF-containing RBC were modelled. First, the possibility was investigated that HbF-containing RBCs release 0, 2, or 16 merozoites (p_f_ = 0, 2, or 16), while HbA-containing RBCs always release 16 (p_a_ = 16). Second, the rate at which infants produce HbF and HbA during the first few months of life was varied (τ_Cf_ =50, 100, or 200; τ_Ca_ =75, 150 or 300). Third, the possibility was examined that *P. falciparum* has no preferential affinity, an increased affinity for HbF-containing RBCs, or an increased affinity for reticulocytes (ζ_Ref_ > ζ_Mf_ and ζ_Rea_ > ζ_Ma_, or ζ_Ref_ > ζ_Rea_ and ζ_Mf_ > ζ_Ma_). Last, the possibility that in HbF-containing RBCs the schizont stage is delayed 60 h (D_Sf_ =72), (so that the life cycle is 108 h instead of 48 h), was modelled. Figure 1 shows the changing rates of parasite population growth under these sets of conditions if an infant is infected at 40 days of age, for example. In these plots, it is clear that if HbF-containing RBCs release fewer merozoites per infected cell, the rate increases more slowly than if they release the same number as an HbA-containing cell. Adding other effects either amplifies (when p_f_ = 0, the infection increases more slowly – graphically, a lower slope) or diminishes this effect (i e, the slope of p_f_ = 0 and p_f_ = 2 approaches that of p_f_ = 16). Adding a delay is mildly protective for the host, as it slightly lowers the parasite population growth rate, which increases the time until host death. This change is seen in Figure 1B as a slightly lower slope in the number of infected RBCs. An increased affinity for HbF is very protective for the host: if an infant is infected at 40 days of age and p_f_ = 0, for instance, as in Figure 1C, the infant actually clears the infection, rapidly. An increased affinity for reticulocytes is harmful for the host: parasite population growth increases more rapidly than if all RBCs are infected at equal rates (Figure 1D compared to Figure 1A).

**Figure 2:**
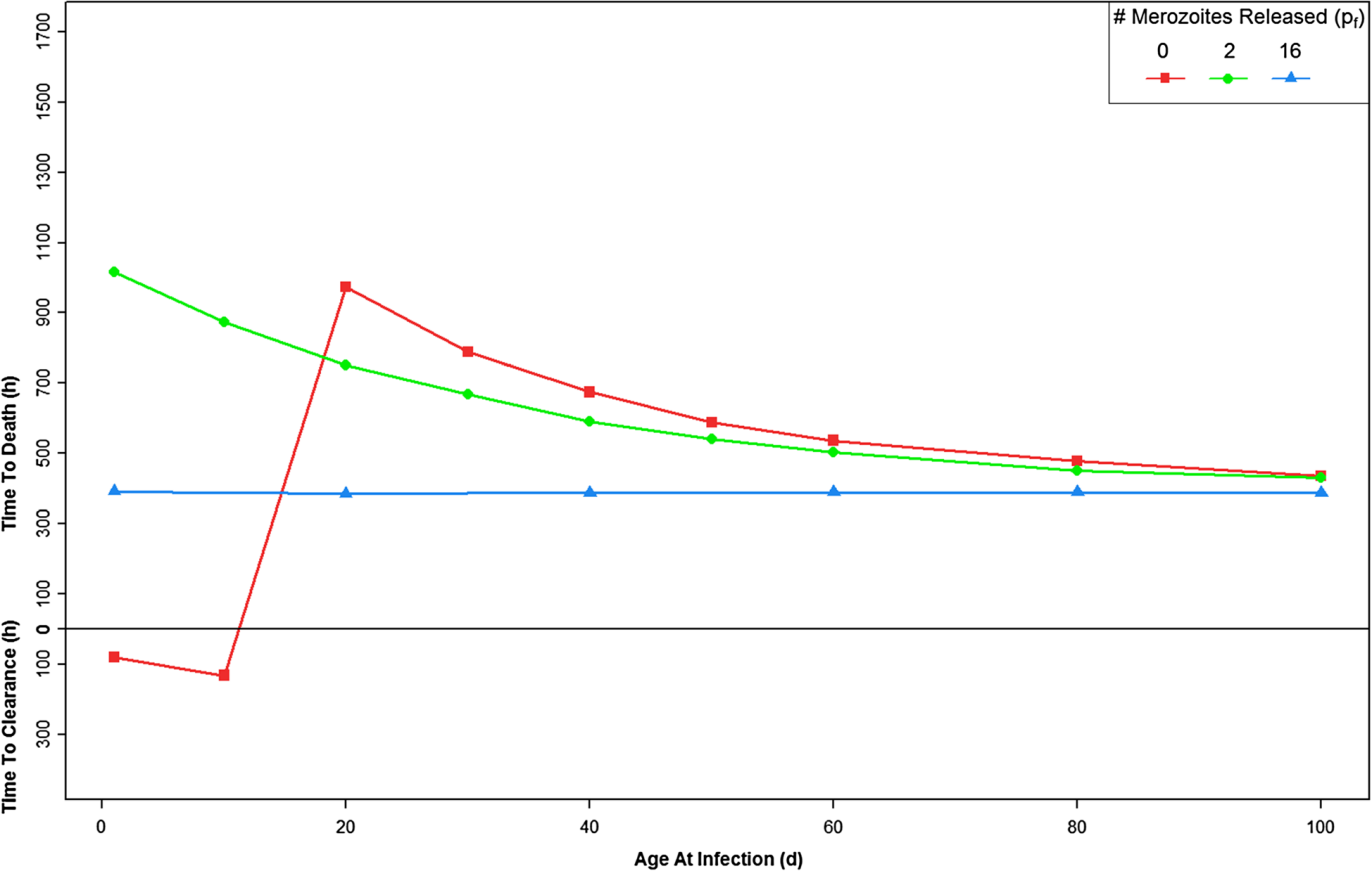
**The time until the host clears infection (y-axis: below h = 0) or the host dies (above h = 0; haematocrit < 24%), when infected, occurs between one and 100 days of age (x-axis).** Parameters set to values in Tables 1 and 2. Results are shown for three different values of *p*_f_, the number of merozoites released per bursting schizont in foetal RBCs. The number of merozoites released from bursting schizonts in adult RBCs, *p*_a_, is 16 for all cases. For more explanation of this figure, see Additional file 2 .

With the parameters set to the values in Tables 1 and 2, the model shows that if HbF-containing RBCs release no new merozoites (p_f_ = 0), the infant remains protected from a fatal outcome of infection through approximately the first 15 days of life, despite the complete absence of an immune response (Figure 2; Additional file 2). If the HbF-containing RBCs release only two merozoites (p_f_ =2), the infant dies but the time until death increases, most significantly in the first two months of life. If the HbF-containing RBCs release 16 merozoites (p_f_ = 16), the same as HbA-containing RBCs, there is no effect; i e, this serves as a control. Now, other parameter modifications will be introduced and compared to this set of parameters.

**Figure 3:**
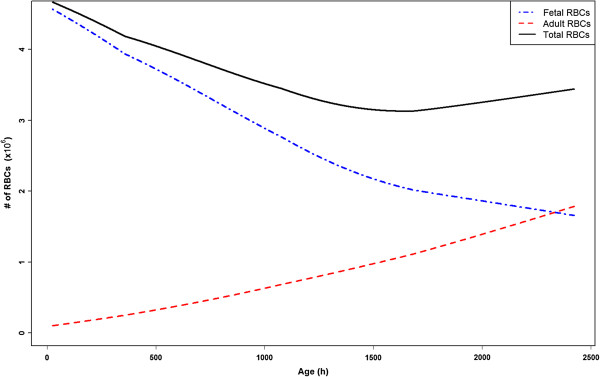
**The number of circulating HbF- and HbA-containing RBCs in the model, in an uninfected infant, with parameters set to the values in Tables**1**and**2 .

**Figure 4:**
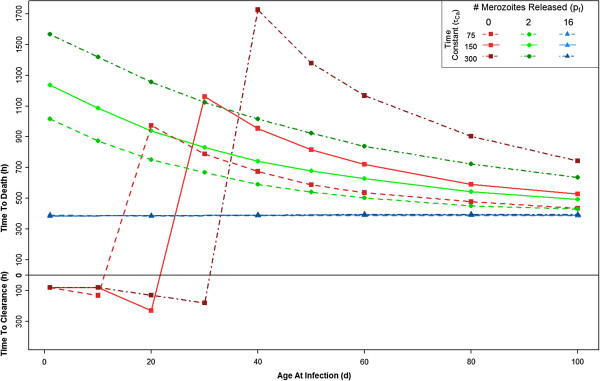
**The effect of different rates of HbF:HbA production.** Three distinct sets of parameters were used: τ_Cf_ =50 and τ_Ca_ =75; τ_Cf_ =100 and τ_Ca_ =150; or τ_Cf_ =200 and τ_Ca_ =300. As expected, when τ_Cf_ and τ_Ca_ are larger (i.e. there is more HbF for longer), the host is protected for a longer period of time. The solid lines are repeated from Figure 2 for comparison.

The rate of change of the ratio of HbF- to HbA-containing RBCs is known to vary dramatically from infant to infant. While some infants reach adult levels of HbA at three months of age, others do not reach adult levels until six months. Three different rates of change were modeled by modifying the values of τ_Cf_ and τ_Ca_, such that the host reaches a HbF:HbA ratio of 1:1 at 65, 97, and 163 days of age. Figure 3 shows the rate of change of HbF- to HbA-containing RBCs when τ_Cf_ = 100, and τ_Ca_ = 150. Figure 4 shows the effect of these rate variations on the infection dynamics across the first 100 days of life. If HbF-containing RBCs release fewer merozoites than HbA-containing RBCs, then the infants with more HbF are more protected. Figure 4 shows that when τ_Cf_ = 200 and τ_Ca_ = 300 the infant is most protected from a fatal outcome.

**Figure 5:**
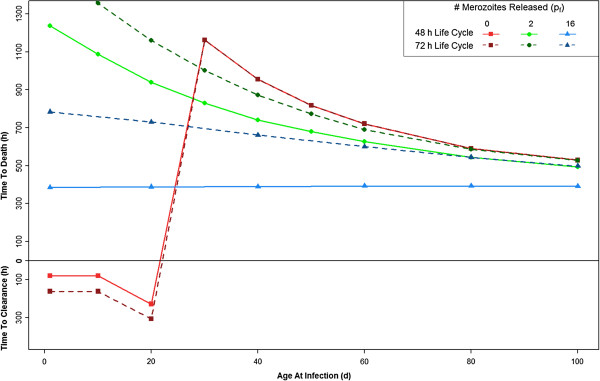
**The time until the host clears infection (y-axis: below h = 0) or the host dies (above h = 0; haematocrit < 24%) when infected between one and 100 days of age (x-axis).** Note that this figure contains the same plot as Figure 2, and the effect of p_f_ = 0, 2, or 16 when there is a delayed, 108 h life cycle (ζ_Ref_ = 10ζ_Rea_ and ζ_Mf_ = 10ζ_Ma_).

In normal conditions, the *P. falciparum* life cycle within a RBC is 48 hours. It is possible that the presence of HbF delays this life cycle. A delay of 60 hours in the schizont phase of HbF-containing RBCs, leading to a total 108-hour life cycle (D_Sf_ =72) was modelled. This delay has little effect if the HbF-containing RBCs act as a “dead end” (0 merozoites released; p_f_ = 0) for the parasite (Figure 5; also Figure 1B) compared to the normal life cycle. If p_f_ =2, and the infant is infected at one day of age, the delay increases the time to host death by about 300 hours. This gain in time to host death slowly decreases as the age of the host at the time of infection increases. Similarly, if p_f_ = 16 (the control) in an infant infected at one day of age, the delay increases the time to host death by 400 hours, a gain which slowly decreases as the age at infection increases.

**Figure 6:**
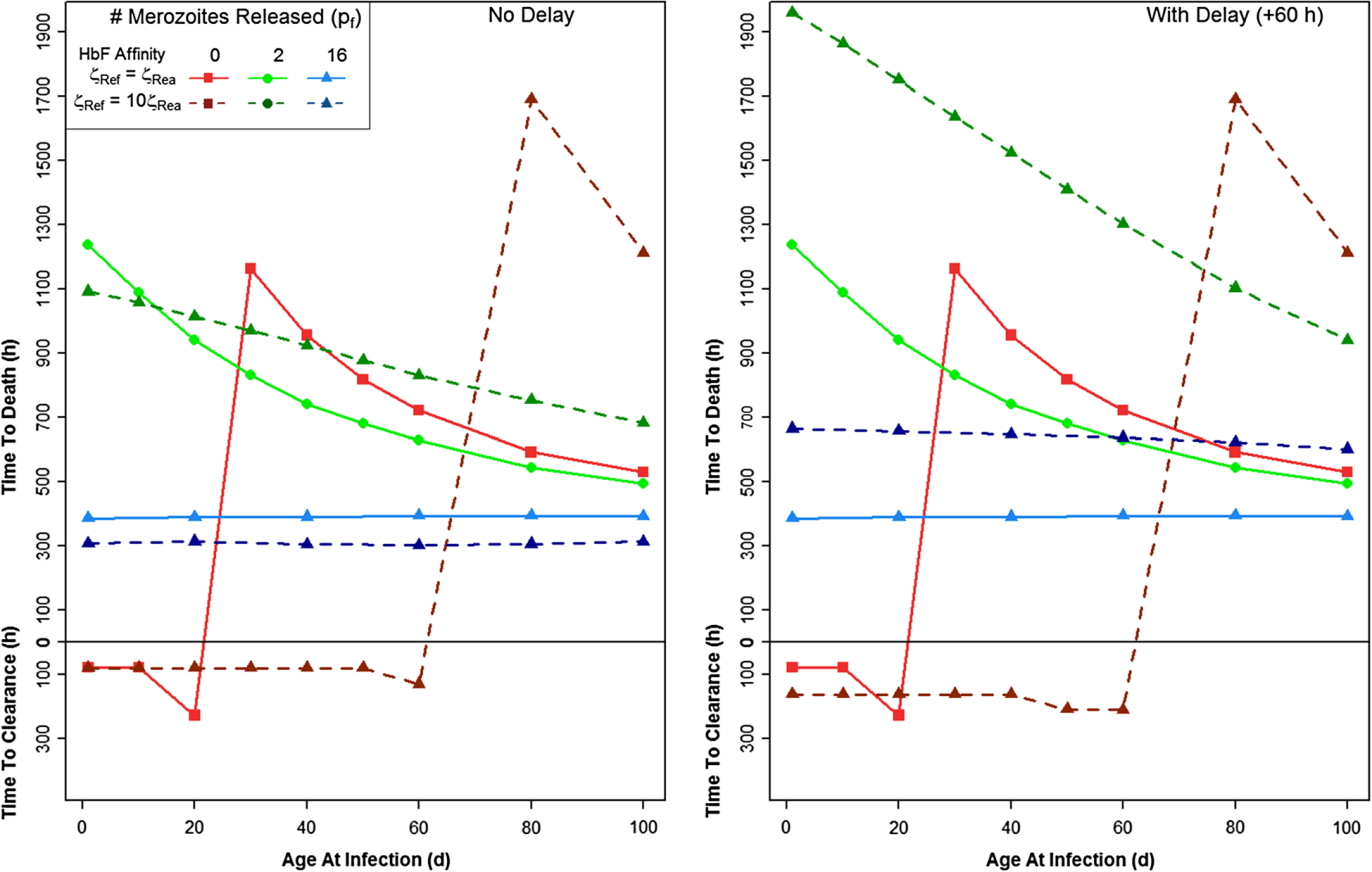
**The time until the host clears infection or the host dies when infected between one and 100 days of age.** In this figure, the parasite has a stronger affinity for HbF-containing RBCs, and either has a normal 48 h life cycle, or a delayed 108 h life cycle (ζ_Ref_ = 10ζ_Rea_ and ζ_Mf_ = 10ζ_Ma_).

Foetal haemoglobin-containing RBCs may be preferentially invaded due only to their younger age distribution, or there may be some inherent HbF-related RBC property for which *P. falciparum* has a greater affinity. This possibility was modelled by increasing the parasite’s affinity for HbF RBCs 10-fold (ζ_Ref_ > ζ_Rea_ and ζ_Mf_ > ζ_Ma_). Compared to ζ_Ref_ = ζ_Rea_ and ζ_Mf_ = ζ_Ma_, the stronger HbF affinity changes infection dynamics in favour of the host, most significantly when p_f_ = 0 (Figure 6). Adding the 60-hour delay to this preferential binding affinity shifts infection dynamics even further in favour of the host, and makes the most significant change in time to host death from the two affinities without delay when p_f_ = 2.

**Figure 7:**
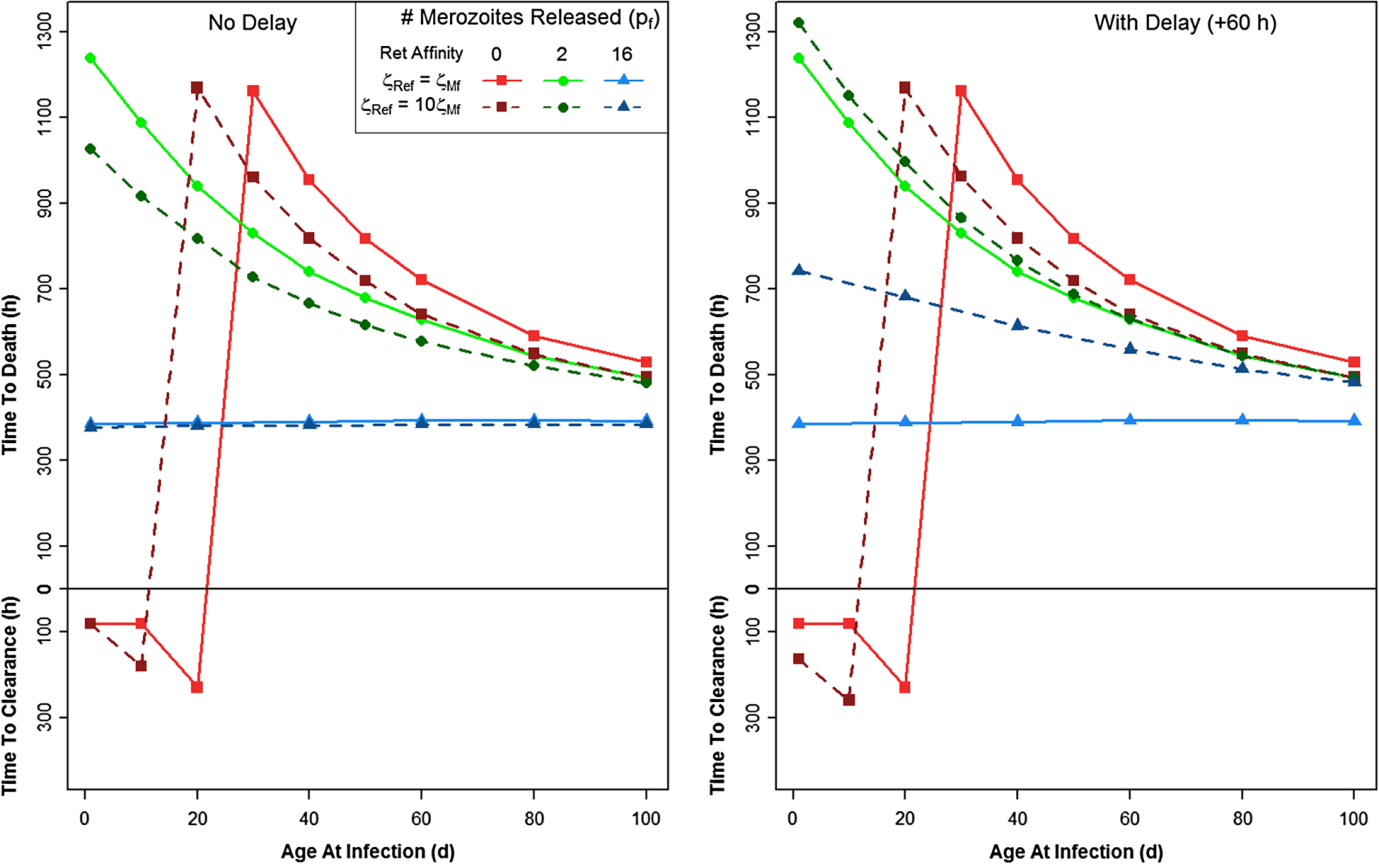
**The time until the host clears infection or the host dies when infected between one and 100 days of age.** In this figure, the parasite has a stronger affinity for reticulocytes, and either has a normal 48 h life cycle, or a delayed 108 h life cycle (ζ_Ref_ = 10ζ_Mf_ and ζ_Rea_ = 10ζ_Ma_).

It has been reported that *P. falciparum* has a higher affinity for reticulocytes than for adult RBCs [30]. This possibility was incorporated into the model by increasing the parasite’s affinity for reticulocytes by 10-fold (ζ_Rea_ > ζ_Ma_ and ζ_Ref_ > ζ_Mf_). An increased preference for reticulocytes slightly changes infection dynamics, although the maximum time to host death does not decrease dramatically (Figure 7). Incorporating a 60-hour delay in merozoite release in this scenario does not appreciably change the effects from those without the delay if p_f_ = 0 or 2. If p_f_ = 16 (the control), in an infant infected at one day of age, the time to host death increases by 400 hours, a gain which slowly decreases as the age at infection increases.

## Discussion

The model presented here incorporates haematological changes that occur in infants under six months of age, and the results may offer some help toward explaining why neonates are relatively protected from severe malaria. The results are in accord with empirical studies which show that the youngest among the age group in question are protected, but the point at which the shift occurs varies based on transmission: in areas of high transmission, infants are protected only through the first three months of life, while in areas of low transmission, infants seem to be protected through six months [36]. It is possible that in areas of high transmission, HbF-containing RBCs are destroyed more quickly as a result of infection and, therefore, the switch to HbA-containing RBCs occurs more quickly. Thus, in areas of high transmission, infants may be protected for a shorter period of time. In a study of three regions of Togo, parasitaemia prevalence increased from 18.2% in infants zero to two months, to 43.0% in infants three to five months. The prevalence of anaemia was also reported to have increased from zero to two months to three to five months of age, with “anaemia more likely in children with parasitaemia.” Overall, there was no significant difference in parasite burden in the different regions, and, in all regions, most clinical disease was observed in infants under two years of age [37]. Another study examined infants younger than three months of age in Gabon, and found a parasite prevalence of 0.1%, concluding that parasitaemia in infants younger than three months of age is very uncommon [38]. A study in Malawi that measured the parasitaemia of all paediatric admissions (<15 yrs) found that 4.8% of positive smears were in children <6 months and 80.7% were in children between six months and five years of age. They concluded that although the risk of infection is lower in children in the younger age group than in older children, the risk does increase in early infancy and that this age group is at a greater risk for infection than previously thought [16].

In the model, when the parameters are set as in Tables 1 and 2, if p_f_ = 0, the host is protected for the first two months of life, despite the complete absence of an immune response. For the next few months of life, the rate at which parasitaemia increases in the course of an infection is slower than in an adult host, and this difference may be sufficient to allow for effective immune intervention: any host factor that slows down parasite population growth buys time for protective responses by maternal antibody or the developing, immature infant immune system. Whatever the mechanism by which HbF retards the expansion of a *Plasmodium* population – whether parasite multiplication in an individual RBC is lower, slower or absent – the presence of HbF would appear to be an unmitigated benefit to the malaria-exposed, of any age. Yet the sparse evidence suggests that HPFH is rare, in areas of high malaria endemicity as elsewhere. Sickle cell anaemia and β-thalassaemia are generally accompanied by elevated HbF levels, but the degree of elevation is highly variable [39,40]. Because elevated HbF levels ameliorate the clinical symptoms of sickle cell disease and β-thalassaemia, and the switch from HbF to HbA production is neither complete nor irreversible, therapeutic reactivation is an active area of research [41]: hydroxyurea is the standard agent in current clinical practice [42]. The potential of these approaches for interventions in malaria-endemic regions apparently remains unexplored [43].

It seems possible that there are opportunities for laboratory researchers to gain important insights from in vitro work. For instance, if sufficient quantities of HbF-containing RBCs could be obtained, and parasite cultures established in them, critical observations could be compared to those from the usual cultures of HbA-containing RBCs, on the number of merozoites released per infected HbF-containing RBC, and the time from when the parasite enters the HbF-containing RBC until it bursts.

The hypothesis that HbF-containing RBCs release fewer merozoites has a greater overall effect on infection dynamics than a delay in the parasite life cycle. Empirical research suggests that the parasite may have a reticulocyte preference, and that this may confound the seeming preference for HbF *in vivo* through the differing age distributions of HbA- and HbF-containing RBCs [19]. Although a reticulocyte preference seems more beneficial to parasite persistence than host survival, in combination with a reduced merozoite release the overall effect still benefits the host.

## Conclusion

It has long been accepted that children under six months of age are protected from severe infection, and this is widely reflected in the literature on paediatric malaria. This topic has recently been analysed more carefully, as noted above, and it appears that the malaria burden in this age group – while still relatively low – may be higher than previously acknowledged [16]. It may be that age-related variation within the overall age group arises from variation in HbF levels and transmission intensity.

The results from this simple model of haematological changes in infants and their effects on *P. falciparum* infection dynamics seem intuitively sound in a qualitative sense, and they emphasize the likely importance of HbF as an explanatory factor in paediatric malaria. The model provides a framework for examining hypotheses about the protective effects of foetal haemoglobin, organizing empirical observations and making critical quantitative comparisons among them.

## Abbreviations

RBC: Red blood cell; *P. falciparum*: *Plasmodium falciparum*; HbF: Foetal haemoglobin; HbA: Adult haemoglobin; HPFH: Hereditary persistence of foetal haemoglobin; ODE: Ordinary differential equation.

## Competing interests

The authors have declared no competing interests.

## Authors' contributions

EB conducted the model runs and compiled the output. All authors contributed to the development of the model and wrote, read and approved the final manuscript.

## Supplementary Material

Additional file 1 Further Methods; Description: Further details on the methods. Click here for file

Additional file 2 **Explanation of Figure 2; Description: Further explanation of the interpretation of Figure**2**.** Click here for file

## References

[B1] WHO World Malaria Report 2010 World Health Organization, Geneva

[B2] Carneiro I Roca-Feltrer A Griffin JT Smith L Tanner M Schellenberg JA Greenwood B Schellenberg D Age-patterns of malaria vary with severity, transmission intensity and seasonality in sub-Saharan Africa: a systematic review and pooled analysis PLoS One 2010 5 e8988 10.1371/journal.pone.0008988 20126547 PMC2813874

[B3] Reyburn H Mbatia R Drakeley C Bruce J Carneiro I Olomi R Cox J Nkya WM Lemnge M Greenwood BM Riley EM Association of transmission intensity and age with clinical manifestations and case fatality of severe Plasmodium falciparum malaria JAMA 2005 293 1461 1470 10.1001/jama.293.12.1461 15784869

[B4] Okiro EA Al-Taiar A Reyburn H Idro R Berkley JA Snow RW Age patterns of severe paediatric malaria and their relationship to Plasmodium falciparum transmission intensity Malar J 2009 8 4 10.1186/1475-2875-8-4 19128453 PMC2630996

[B5] Baird JK Age-dependent characteristics of protection v. susceptibility to Plasmodium falciparum Ann Trop Med Parasitol 1998 92 367 390 10.1080/00034989859366 9683890

[B6] Billig E O'Meara W Riley E McKenzie FE Developmental allometry and paediatric malaria Malar J 2012 11 64 10.1186/1475-2875-11-64 22394452 PMC3331816

[B7] Miller LH Good MF Milon G Malaria pathogenesis Science 1878 1994 264 10.1126/science.8009217 8009217

[B8] Raes A Van Aken S Craen M Donckerwolcke R Vande Walle J A reference frame for blood volume in children and adolescents BMC Pediatr 2006 6 3 10.1186/1471-2431-6-3 16503982 PMC1434736

[B9] Boer P Estimated lean body mass as an index for normalization of body fluid volumes in humans Am J Physiol 1984 247 F632 636 6496691 10.1152/ajprenal.1984.247.4.F632

[B10] Sankaran VG Xu J Orkin SH Advances in the understanding of haemoglobin switching British J Haematol 2010 149 181 194 10.1111/j.1365-2141.2010.08105.x PMC4153468 20201948

[B11] Cook CD Brodie HR Allen DW Measurement of fetal hemoglobin in newborn infants Pediatrics 1957 20 272 278 13452668

[B12] Stamatoyannopoulos G Molecular and cellular basis of hemoglobin switching Disorders of hemoglobin: Genetics, pathophysiology, and clinical management 2001

[B13] Karlsson S Nienhuis AW Developmental regulation of human globin genes Ann Rev Biochem 1985 54 1071 1108 10.1146/annurev.bi.54.070185.005231 2411209

[B14] Forget BG Progress in understanding the hemoglobin switch N Engl J Med 2011 365 852 854 10.1056/NEJMe1106969 21879905

[B15] Oneal PA Gantt NM Schwartz JD Bhanu NV Lee YT Moroney JW Reed CH Schechter AN Luban NL Miller JL Fetal hemoglobin silencing in humans Blood 2006 108 2081 2086 10.1182/blood-2006-04-015859 16735596 PMC1895549

[B16] Larru B Molyneux E ter Kuile F Taylor T Molyneux M Terlouw D Malaria in infants below six months of age: retrospective surveillance of hospital admission records in Blantyre, Malawi Malar J 2009 8 310 10.1186/1475-2875-8-310 20038299 PMC2805692

[B17] Riley E Wagner G Ofori M Wheeler J Akanmori B Tetteh K McGuinness D Bennett S Nkrumah F Anders R Lack of association between maternal antibody and protection of African infants from malaria infection Infect Immun 2000 68 5856 5863 10.1128/IAI.68.10.5856-5863.2000 10992495 PMC101547

[B18] Nagel RL Innate resistance to malaria: the intraerythrocytic cycle Blood Cells 1990 16 321 339 discussion 340–329 2257317

[B19] Pasvol G Weatherall DJ Wilson RJM Effects of foetal haemoglobin on susceptibility of red cells to Plasmodium falciparum Nature 1977 270 171 173 10.1038/270171a0 337159

[B20] Pasvol G Wilson RJ The interaction of malaria parasites with red blood cells Br Med Bull 1982 38 133 140 7052193 10.1093/oxfordjournals.bmb.a071749

[B21] Pasvol G Weatherall DJ Wilson RJ Smith DH Gilles HM Fetal haemoglobin and malaria Lancet 1976 1 1269 1272 73695 10.1016/s0140-6736(76)91738-4

[B22] Wilson RJ Pasvol G Weatherall DJ Invasion and growth of Plasmodium falciparum in different types of human erythrocyte Bull World Health Organ 1977 55 179 186 338178 PMC2366726

[B23] Amaratunga C Lopera-Mesa TM Brittain NJ Cholera R Arie T Fujioka H Keefer JR Fairhurst RM A role for fetal hemoglobin and maternal immune IgG in infant resistance to Plasmodium falciparum malaria PLoS One 2011 6 e14798 10.1371/journal.pone.0014798 21532754 PMC3075246

[B24] McQueen PG McKenzie FE Age-structured red blood cell susceptibility and the dynamics of malaria infections Proc Natl Acad Sci U S A 2004 101 9161 9166 10.1073/pnas.0308256101 15178766 PMC428490

[B25] McQueen PG McKenzie FE Competition for red blood cells can enhance Plasmodium vivax parasitemia in mixed-species malaria infections Am J Trop Med Hyg 2006 75 112 125 16837717 PMC2483695

[B26] McQueen PG McKenzie FE Host control of malaria infections: constraints on immune and erythropoeitic response kinetics PLoS Comput Biol 2008 4 e1000149 10.1371/journal.pcbi.1000149 18725923 PMC2491590

[B27] Lloyd AL Destabilization of epidemic models with the inclusion of realistic distributions of infectious periods Proc Biol Sci 2001 268 985 993 10.1098/rspb.2001.1599 11370974 PMC1088698

[B28] Pearson HA Life-span of the fetal red blood cell J Pediatr 1967 70 166 171 10.1016/S0022-3476(67)80410-4 5334979

[B29] Yamashita H Kukita J Ohga S Nakayama H Akazawa K Ueda K Serum erythropoietin levels in term and preterm infants during the first year of life J Pediatr Hematol Oncol 1994 16 213 10.1097/00043426-199408000-00005 8037338

[B30] Pasvol G Weatherall DJ Wilson RJM The increased susceptibility of young red cells to invasion by the malarial parasite Plasmodium falciparum Brit J Haematol 1980 45 285 295 10.1111/j.1365-2141.1980.tb07148.x 7002199

[B31] Baer K Klotz C Kappe SHI Schnieder T Frevert U Release of hepatic Plasmodium yoelii merozoites into the pulmonary microvasculature PLoS Pathog 2007 3 e171 10.1371/journal.ppat.0030171 17997605 PMC2065874

[B32] Glushakova S Yin D Li T Zimmerberg J Membrane transformation during malaria parasite release from human red blood cells Curr Biol 2005 15 1645 1650 10.1016/j.cub.2005.07.067 16169486

[B33] McKenzie FE Jeffery GM Collins WE Plasmodium vivax blood-stage dymanics J Parasitol 2002 88 521 535 12099421 10.1645/0022-3395(2002)088[0521:PVBSD]2.0.CO;2 PMC2504326

[B34] Collins W Jeffery G A retrospective examination of the patterns of recrudescence in patients infected with Plasmodium falciparum Am J Trop Med Hyg 1999 61 44 48 10432044 10.4269/tropmed.1999.61-044

[B35] Hamilton Fairley BN Sidelights on malaria in man obtained by subinoculation experiments Trans R Soc Trop Med Hyg 1947 40 621 676 10.1016/0035-9203(47)90025-4 20243883

[B36] Snow RW Nahlen B Palmer A Donnelly CA Gupta S Marsh K Risk of severe malaria among African infants: direct evidence of clinical protection during early infancy J Infect Dis 1998 177 819 822 10.1086/517818 9498474

[B37] Eliades MJ Wolkon A Morgah K Crawford SB Dorkenoo A Sodahlon Y Hawley WA Hightower AW Kuile FOT Terlouw DJ Burden of malaria at community level in children less than 5 years of age in Togo Am J Trop Med Hyg 2006 75 622 629 17038683

[B38] Klein Klouwenberg PMC Oyakhirome S Schwarz NG Gläser B Issifou S Kiessling G Klöpfer A Kremsner PG Längin M Lassmann B Malaria and asymptomatic parasitaemia in Gabonese infants under the age of 3 months Acta Trop 2005 95 81 85 10.1016/j.actatropica.2005.05.003 15950165

[B39] Weatherall DJ Phenotype-genotype relationships in monogenic disease: lessons from the thalassaemias Nat Rev Genet 2001 2 245 255 10.1038/35066048 11283697

[B40] Thein SL Menzel S Lathrop M Garner C Control of fetal hemoglobin: new insights emerging from genomics and clinical implications Hum Mol Gen 2009 18 R216 R223 10.1093/hmg/ddp401 19808799 PMC2758709

[B41] Wilber A Nienhuis AW Persons DA Transcriptional regulation of fetal to adult hemoglobin switching: new therapeutic opportunities Blood 2011 117 3945 3953 10.1182/blood-2010-11-316893 21321359 PMC3087525

[B42] McGann PT Ware RE Hydroxyurea for sickle cell anemia: what have we learned and what questions still remain? Curr Opin Hematol 2011 18 158 165 10.1097/MOH.0b013e32834521dd 21372708 PMC3181131

[B43] Makani J Williams TN Marsh K Sickle cell disease in Africa: burden and research priorities Ann Trop Med Parasitol 2007 101 3 14 10.1179/136485907X154638 17244405 PMC5612390

[B44] Miller MW Brayman AA Sherman TA Abramowicz JS Cox C Comparative sensitivity of human fetal and adult erythrocytes to hemolysis by pulsed 1 MHz ultrasound Ultrasound Med Biol 2001 27 419 425 10.1016/S0301-5629(00)00350-1 11369128

[B45] White JBN AS Fauci EB, Kasper DL Malaria Harrison's Principles of Internal Medicine 2008 McGraw-Hill, New York

[B46] Johnson JG Epstein N Shiroishi T Miller LH Factors affecting the ability of isolated Plasmodium knowlesi merozoites to attach to and invade erythrocytes Parasitology 1980 80 539 550 10.1017/S0031182000000998 6771738

[B47] Rapaport S Introduction to Hematology 1987 J.B. Lippincott, Philadelphia

