# Supplementary material for: Foetal haemoglobin and the dynamics of paediatric malaria
Source: Malar J. 2012 Nov 28;11:396. doi: 10.1186/1475-2875-11-396 (PMC3538578; doi:10.1186/1475-2875-11-396)
Supplement: Additional file 1 — Further Methods; Description: Further details on the methods. [file 1475-2875-11-396-S1.doc]

**Mathematical Preliminaries**

Members of a population of a cell type will age unevenly, with some finite lifespan, so to incorporate aging we use a numerically tractable idea adapted by Lloyd from ecology that uses a special structure of compartmental ordinary differential equations [1-2]. This method is used in the simulation of within-host asexual *Plasmodium* population dynamics [3-6].

**Matrix and Vector Notation**

Recall that an *N X M* matrix **A** is a rectangular array of numbers with *N* rows and *M* columns. The components on the ith row and jth column we label as **A**_i,j_ We define matrix addition and multiplication as follows: if two matrices **A** and **B** are *N X M*, then **C** = **A** + **B** is an *N X M* matrix such that

**C**_i,j_ = **A**_i,j_ + **B**_i_,*_j_*

If matrix **A** is *N X P* and matrix **B** is *P X M*, then **C** = **A** **B** is a *N X M* matrix such that

**C**_i,j_ = Σ _k=1,P_ **A**_i,k_ **B**_k,j_

A special class of matrices consists of those of dimensions *N X 1*, called vectors in this report.

Three prominent matrices important in the specification of the dynamical equations are the *N X 1* vector **δ**(n), *N X N* identity matrix ***I*** and the *N X N* “difference'' matrix ***D***:

**δ**(n)_i_ = 1, i = n

= 0 otherwise.

***I***_i,j_ = 1, i = j

= 0 otherwise.

***D***_i,j_ = +1, i = j

= -1, i = j + 1,

= 0 otherwise.

If **V** is a *N X 1* vector, we define *L(***V***)* and *T(***V***)* so that

*L(***V**) = **V**_N_ (i.e., the Nth element of **V**)

*T(***V**) = Σ _i = 1,N_ **V**_i_

Although not a matrix symbol, we define Θ(x) so that

Θ(x) = 1, x > 0,

= 0, x < 0.

**Time Evolution of Mass and Blood Volume of the Host**

For a given time *t* since birth, we computed *W(t)* using a linear interpolation scheme using data points extracted from the weight-for-age tables for boys compiled by the World Health Organization, and from growth charts for boys compiled by the Centers for Disease Control [7-8]. The data points are listed in **Table S1**.

The blood volume per mass of a host as a function of age, *BVPM(t)*, has been measured for humans, [9] so to estimate this quantity we used a linear interpolation of measured data points. The data points are listed in **Table S2**. Then the volume of the blood, *V*_B_*(t)*, is

*V*_B_*(t)* = *W(t) X BVPM(t)*

**Numerical Implementation of the Model**

Equations (4) and (5) describe a coupled system of ordinary differential equations. We used the fifth order Runge- Kutta-Fehlberg algorithm with adaptive time stepping to solve this system [10]. All coding was done in C++. We found that despite the gigantic number of dependent variables in time, we could readily calculate the solution to a precision of one part in 10^6^**.**  Simulations were started with the host at birth, with the weight, blood volume, and fetal and adult red blood cell populations evolving, and continued until after primary release when the host died or all parasites were cleared. The erythrocyte source terms *ESa(t)* *and ESf(t),* as well as *W(t)*, *V*_B_*(t)*, and d*V*_B_*(t )*/dt were updated once every 24 hours of simulated time. If during any time the population per unit volume for those cell types listed in **Table 2 in main text** became less than *V*_B_*(t)*^-1^, all the components of the state vector of that population were set to zero.

The initial conditions were set such that all parasite populations were zero. Also, the initial state vectors for the fetal and adult red blood cell populations were set so that *T(***Rea***)*, *T(***Ma***)*, *T(***Ref***)* and *T(***Mf***)* changed smoothly for all *t* with the forms of *ESa(t)* and *ESf(t)* used above.

**Tables**

**Table S1:** Data Points Used to Interpolate Host Mass as a Function of Age

| Age in days | Mass in kg |
| --- | --- |
| 0.0 | 3.346 |
| 70.0 | 5.835 |
| 120.0 | 6.97 |
| 180.0 | 7.9 |
| 300.0 | 9.128 |
| 2008.0 | 19.53 |
| 3042.0 | 26.56 |
| 4745.0 | 45.59 |
| 5551.0 | 57.32 |
| 6083.0 | 63.47 |
| 7072.0 | 69.74 |
| 7315.0 | 70.64 |

**Table S2: Data Points Used to Interpolate Blood Volume per Host Mass as a Function of Age**

| Age in days | Volume per mass  in μl kg ^-1^ |
| --- | --- |
| 0 | 81900.0 |
| 15.0 | 84400.0 |
| 45.0 | 79400.0 |
| 135.0 | 76600.0 |
| 285.0 | 82400.0 |
| 555.0 | 86100.0 |
| 547.5 | 80500.0 |
| 1825.0 | 76700.0 |
| 3102.5 | 79600.0 |
| 4562.5 | 74400.0 |
| 7300.0 | 71428.5 |

**References**

1. Lloyd AL: **Destabilization of epidemic models with the inclusion of realistic distributions of infectious periods.** *Proc Biol Sci* 2001, **268:**985-993.

2. Lloyd AL: **The dependence of viral parameter estimates on the assumed viral life cycle: limitations of studies of viral load data.** *Proc Biol Sci* 2001, **268:**847-854.

3. McQueen PG, McKenzie FE: **Age-structured red blood cell susceptibility and the dynamics of malaria infections.** *Proc Natl Acad Sci U S A* 2004, **101:**9161-9166.

4. McQueen PG, McKenzie FE: **Host control of malaria infections: constraints on immune and erythropoeitic response kinetics.** *PLoS Comput Biol* 2008, **4:**e1000149.

5. McQueen PG, McKenzie FE: **Competition for red blood cells can enhance Plasmodium vivax parasitemia in mixed-species malaria infections.** *Am J Trop Med Hyg* 2006, **75:**112-125.

6. McQueen PG: **Population dynamics of a pathogen: the conundrum of vivax malaria.** *Biophys Rev* 2010, **2:**111-120.

7. Organization WH: **WHO Child Growth Standards.** In *Book WHO Child Growth Standards* 2012.

8. Control CfD: **CDC Growth Charts.** In *Book CDC Growth Charts* 2010.

9. Linderkamp O, Versmold HT, Riegel KP, Betke K: **Estimation and prediction of blood volume in infants and children.** *Eur J Pediatr* 1977, **125:**227-234.

10. Cash JR KA: **A variable order Runge-Kutta method for initial value problems with rapidly varying right-hand sides.** *Association for Computing Machinery Transactions on Mathematical Software* 1990, **16:**201-222.
