# Supplementary material for: Foetal haemoglobin and the dynamics of paediatric malaria
Source: Malar J. 2012 Nov 28;11:396. doi: 10.1186/1475-2875-11-396 (PMC3538578; doi:10.1186/1475-2875-11-396)
Supplement: Additional file 2 — Explanation of Figure 2; Description: Further explanation of the interpretation of Figure2. [file 1475-2875-11-396-S2.docx]

**Explanation of Figure 2**:

Scenario 1 (blue): When 16 merozoites are released per infected fetal RBC, the fate of the host is always death – never parasite clearance – and the time to death does not vary with host age at infection. This result is expected because in this case because HbF-containing RBCs behave the same as HbA-containing RBCs infected with a parasite.

Scenario 2 (green): When 2 merozoites are released per infected fetal RBC, the time until host death is higher for younger ages (ie. the line slopes downward, asymptotically approaching the time until host death when 16 merozoites are released).

Scenario 3 (red): When an infected HbF-containing RBC is a dead-end for the parasite (ie. does not release any merozoites) for very young infants, the parasite population dies out (the y-value is below 0). This clearance occurs without an immune response. It is due to the high proportion of HbF-containing RBCs in the host. In this scenario, when the host is infected after ~15 days of age (when the y=0) the HbF-containing RBCs can no longer clear the parasite population without the help of the immune system. From this point forward, the infant dies without any immune response*.*

Note that for *p*_f_ = 0 and *p*_f_ = 2, the curves showing time to host death as a function of age of infection approaches asymptotically the curve for *p*_f_ = 16 as the ratio of HbF to HbA RBCs stabilizes and HbA RBCs start to dominate the RBC population.

These 3 scenarios (blue, green, and red curves) apply to Figures 4-7 as well as Figure 2. Additional curves in Figures 4-7 represent the additional factors noted in the corresponding figure legends and text.
